# Gasless laparoscopy in rural India-registry outcomes and evaluation of the learning curve

**DOI:** 10.1007/s00464-023-10392-4

**Published:** 2023-08-31

**Authors:** N. Aruparayil, J. Gnanaraj, A. Mishra, L. Bains, N. Corrigan, J. Brown, T. Ensor, R. King, B. Shinkins, D. Jayne

**Affiliations:** 1 Leeds Institute of Medical Research, University of Leeds, Leeds, UK; 2 Karunya University, Coimbatore, India; 3 Maulana Azad Medical College, Delhi, India; 4 Leeds Institute of Clinical Trials Research, University of Leeds, Leeds, UK; 5 Nuffield Centre for International Health and Development, University of Leeds, Leeds, UK; 6 Academic Unit of Health Economics, University of Leeds, Leeds, UK; 7 Division of Health Sciences, University of Warwick, Coventry, UK; 8 St. James’s University Hospital, Level 7, Clinical Sciences Building, Leeds, LS9 7TF UK

**Keywords:** Gasless laparoscopy, Abdominal wall lift, CUSUM, Learning curve, Training, Rural surgery

## Abstract

**Background:**

A program of gasless laparoscopy (GL) has been implemented in rural North-East India. To facilitate safe adoption, participating rural surgeons underwent rigorous training prior to independent clinical practice. An online registry was established to capture clinical data on safety and efficacy and to evaluate initial learning curves for gasless laparoscopy.

**Methods:**

Surgeons who had completed the GL training program participated in the online RedCap Registry. Patients included in the registry provided informed consent for the use of their data. Data on operative times, conversion rates, perioperative complications, length of stay, and hospital costs were collected. Fixed reference cumulative sum (CUSUM) model was used to evaluate the learning curve based on operative times and conversion rates published in the literature.

**Results:**

Four surgeons from three rural hospitals in North-East India participated in the registry. The data were collected over 12 months, from September 2019 to August 2020. One hundred and twenty-three participants underwent GL procedures, including 109 females (88.6%) and 14 males. GL procedures included cholecystectomy, appendicectomy, tubal ligation, ovarian cystectomy, diagnostic laparoscopy, and adhesiolysis. The mean operative time was 75.3 (42.05) minutes for all the surgeries. Conversion from GL to open surgery occurred in 11.4% of participants, with 8.9% converted to conventional laparoscopy. The main reasons for conversion were the inability to secure an operative view, lack of operating space, and adhesions. The mean length of stay was 3 (2.1) days. The complication rate was 5.7%, with one postoperative death. The CUSUM analysis for GL cholecystectomy showed a longer learning curve for operative time and few conversions. The learning curve for GL tubal ligation was relatively shorter.

**Conclusion:**

Gasless laparoscopy can be safely implemented in the rural settings of Northeast India with appropriate training programs. Careful case selection is essential during the early stages of the surgical learning curve.

**Supplementary Information:**

The online version contains supplementary material available at 10.1007/s00464-023-10392-4.

More than 90% of the global population lacks ready access to surgery. For populations in South Asia, this figure surpasses 95%. In North East India, an estimated 2.4 million surgical procedures per year or 5500 per 100,000 population, are needed [1]. Over 300,000 procedures (one-eighth) each year are amenable to laparoscopic surgery [2]. The benefits of laparoscopic surgery compared to open surgery include a reduction in the rate of negative laparotomies, quicker recovery, reduced pain, shortened hospital stay, and cost savings [3]. In low and middle-income countries (LMICs), the use of laparoscopic techniques raises many challenges, particularly in rural settings. Poor infrastructure, lack of equipment, training, and out-of-pocket expenses often make laparoscopic surgery inaccessible [4–6].

Gasless laparoscopy (GL) has recently attracted attention in LMICs as a potential stepping stone and cost-effective alternative to conventional laparoscopy [7]. It involves attaching a metal arm to the operating table to hold a metal helix ring which is inserted into the abdomen to lift the abdominal wall. Retraction of the abdominal wall creates an operating space using atmospheric air without the need for carbon dioxide insufflation. The procedure can also be performed under spinal rather than general anesthesia [8] (Fig. 1).

**Fig. 1:**
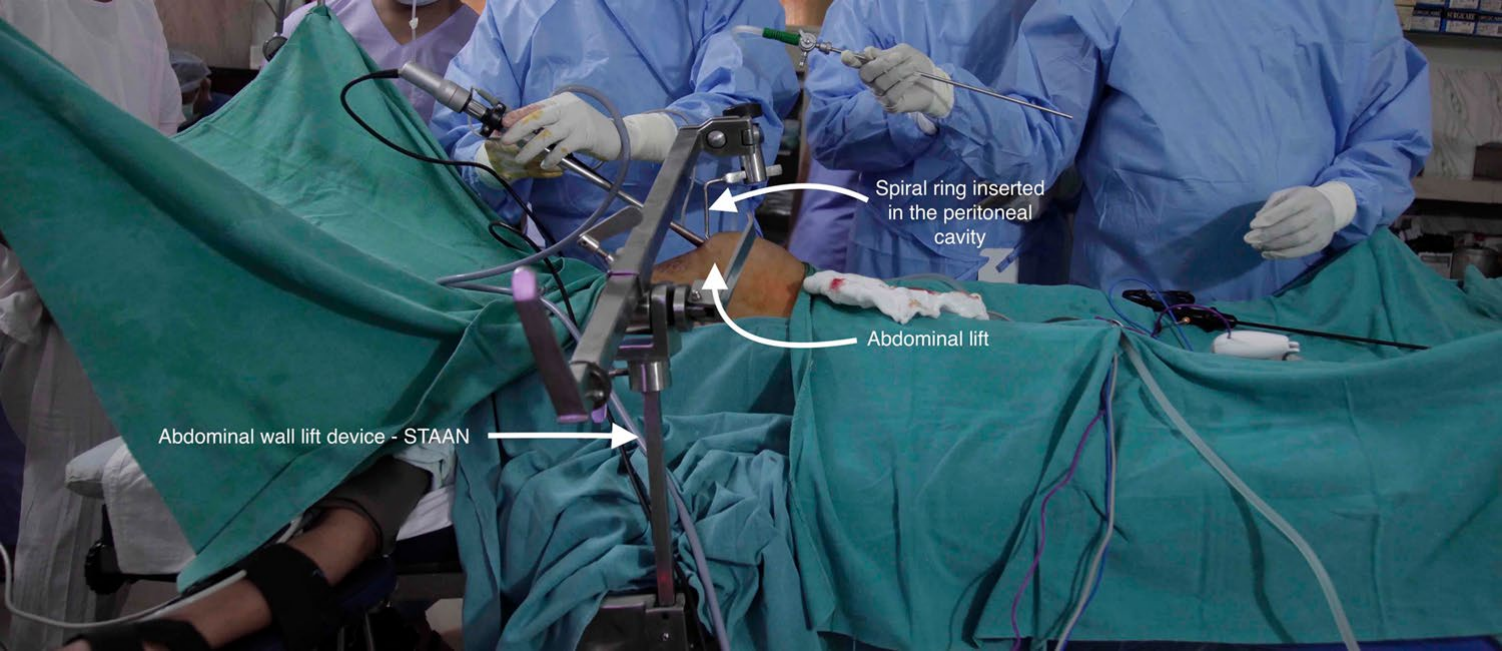
Abdominal wall lift—STAAN device

The TARGET training and proctorship program was established to address the deficit of laparoscopic surgery in rural North-East India, providing structured training opportunities and facilitating the scale-up of gasless laparoscopic [9, 10]. Research suggests that where gasless laparoscopy has been implemented in LMICs, it is cost-effective, safe, and with good clinical outcomes [7, 11–13]. However, few studies have evaluated gasless laparoscopy within a rural setting. In rural settings, there is a shortage of standardized data reporting surgical outcomes due to a lack of resources and infrastructure [14]. Mandavia et al*.* outlined the role of registry data as a valuable alternative to randomized trials, developing a framework to describe the critical components of a successful surgical registry [15]. Based on these recommendations, an online registry was established to assess the safety and efficacy of GL undertaken by rural surgeons in Northeast India and allow a preliminary analysis of learning curves.

## Methods

**Fig. 2:**
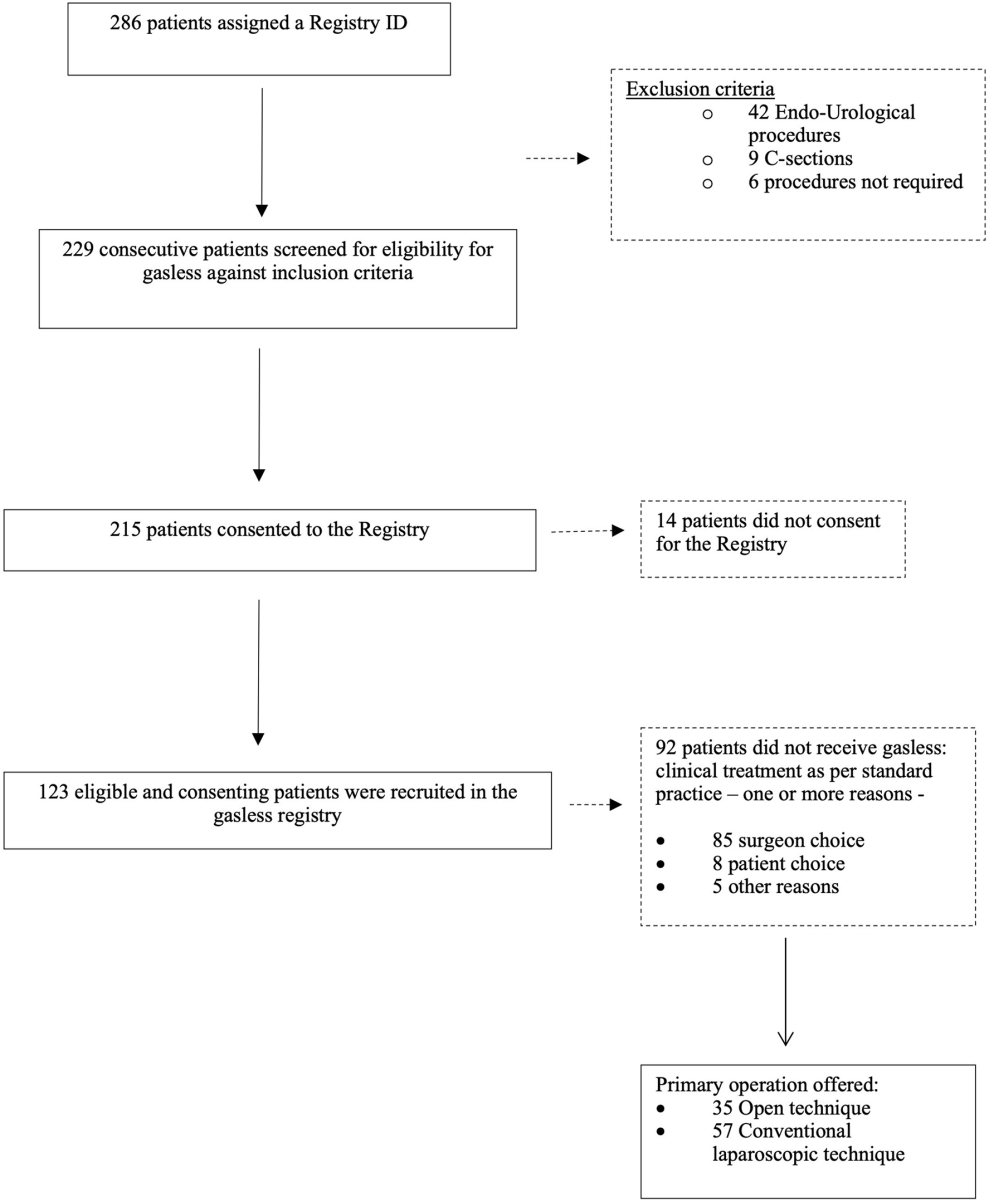
Flowchart of patient recruitment in the gasless registry

All GL operations included in the registry were performed by rural surgeons after training and proctorship in GL and assessment of competency for independent practice [9]. Patients considered eligible for inclusion for GL procedures provided informed consent for the use of their data. GL procedures included cholecystectomy, appendicectomy, tubal ligation, ovarian cystectomy, diagnostic laparoscopy, and adhesiolysis. Figure 2 depicts the schema for patient inclusion in the registry. The registry was a prospective, observational study, and was not powered to evaluate a pre-specified outcome. Statistical analysis was conducted using SPSS (Version 27.0. Armonk, NY: IBM Corp) and Microsoft Excel (2022 version 16.63.1). Ethical approval was obtained from the School of Medicine Research Ethics Committee, University of Leeds (MREC 18–100) and the ethical committee of the Martin Luther Christian University (MLCU), India (VI/I(8)/UREC/EA/272/2015–6111) for three healthcare facilities in Assam, Nagaland, and Manipur.

Complications were classified according to European Perioperative Clinical Outcomes (EPCO) definitions and were graded as mild, moderate, or severe [16]. Patient costs were summarized descriptively to inform trends in clinical practice and decision-making, identify unmet needs for laparoscopic surgery, and determine the feasibility of gasless laparoscopy. A cumulative sum (CUSUM) method was used to assess the learning curve of GL, plotting the values by cumulatively summing the deviations of the observations from fixed reference values published in the literature and mean scores of all values [17, 18]. A CUSUM learning curve (LC) was created for the operative time. A fixed reference point was used to compare surgeon performance to expert-level proficiency. For each surgeon, the LC CUSUM values after the surgeon’s *i*-th operation $\left(i=\mathrm{1,2},\dots ,n\right)$, denoted ${S}_{i}$, was defined as ${S}_{i}=\sum_{j=1}^{i}\left({Y}_{j}-Y\right),$ where ${Y}_{j}$ is the observed operative time in minutes for operation $j$,and $Y$ is a fixed reference value. For operative times, a fixed reference value of the published mean operative time for gasless cholecystectomy (82 min) and tubal ligation (43.5 min) was used [19, 20].

To evaluate CUSUM LC for conversion from gasless laparoscopy to conventional laparoscopy or open surgery for each surgeon, the CUSUM conversion score after the surgeon’s *i*-th operation $\left(i=\mathrm{1,2},\dots ,n\right)$, denoted ${C}_{i}$, was defined as ${C}_{i}=\sum_{j=1}^{i}\left({Y}_{j}-P\right)$, where $P$ is the fixed reference for conversion rate, and ${Y}_{j}$ is a binary indicator for conversion, i.e., is equal to 0 if there is no conversion or 1 if converted to open or conventional laparoscopy. A fixed reference conversion rate of 7% was used [7].

## Results

There were 286 patients assigned a gasless registry ID, and following initial screening and exclusion, a total of 123 patients were included in the gasless registry, as shown in Fig. 2. Four surgeons from three rural hospitals in North-East India (Assam, Nagaland, and Manipur) participated in the study. Each surgeon was given a specific code with the number of procedures performed prior to GL training: surgeon 2—in Nagaland performed 100 laparoscopic procedures; Surgeon 4 performed 300 and Surgeon 5 performed 150 laparoscopic procedures—in Manipur region, and Surgeon 6 in Assam performed 10 laparoscopic procedures [9]. The demographics in Table 1 show that the mean age of the patients was 35.51 years (sd = 11.17), with the majority being female (*n* = 109/123, 88.6%). Overall, 64 (54.4%) patients had a normal BMI, and 48 (39%) were overweight classified, according to the World Health Organization. Most patients were ASA I. A medically qualified anesthetist was present for 75 (61%) cases. The mean length of hospital stay was 3 days (2.1). The mean time to set up the gasless abdominal lift device was 7.41 (3.01) minutes. The mean operative time was 75.3 min (42.05).

**Table 1:** Patient and operative characteristics data are *n (%)* unless stated otherwise. Mean and SD (Standard deviation)

	Assam (SPF)	Manipur (PF)	Nagaland (CHC)	Total
Patients	52	27	44	123
Age				
Mean (SD)	36 (10)	39 (16)	30 (6)	35.51 (11.17)
Sex				
Male	6 (11.5)	7 (25.9)	1 (2.3)	14 (11.4)
Female	46 (88.5)	20 (74.1)	43 (97.9)	109 (88.6)
BMI				
18–24	27 (51.9)	17 (63)	23 (53.3)	67 (54.5)
25–29	21 (40.4)	6 (22.2)	21 (47.7)	48 (39)
30–34	1 (1.9)	4 (14.8)	0 (0)	5 (4.1)
Less than 18	3 (5.8)	0 (0)	0 (0)	3 2.4)
ASA				
I	48 (92.3)	27 (100)	44 (100)	119 (96.7)
II	4 (7.7)	0 (0)	0 (0)	4 (3.3)
Imaging				
X-ray	2 (3.8)	5 (18.5)	4 (9)	11 (8.9)
US	50 (96.1)	27 (100)	44 (100)	121 (98.3)
CT	1 (1.9)	0	0	1 (0.8)
MRI	1 (1.9)	0	0	1 (0.8)
Operation				
Cholecystectomy	39 (75)	14 (51.9)	1 (2.3)	54 (43.9)
Tubal ligation	0 (0)	0 (0)	30 (68.2)	30 (24.3)
Appendicectomy	6 (11.4)	8 (29.6)	6 (13.6)	20 (16.3)
Ovarian cystectomy	4 (7.7)	2 (7.4)	1 (2.3)	7 (5.7)
Other	2 (3.8)	1 (3.7)	3 (6.8)	6 (4.9)
Diagnostic laparoscopy	1 (1.9)	2 (7.4)	2 (4.5)	5 (4.1)
Adhesiolysis	0 (0)	0 (0)	1 (2.3)	1 (0.8)
Type of anesthetist				
Medically qualified specialist	47 (90.4)	27 (100)	1 (2.3)	75 (61)
Medically qualified non-specialist	0 (0)	0 (0)	28 (63.6)	28 (22.8)
No anesthetist	1 (1.9)	0 (0)	2 (4.5)	3 (2.4)
Non doctor anesthetist specialist	0 (0)	0 (0)	10 (22.7)	10 (8.1)
Non doctor and non-specialist	4 (7.7)	0 (0)	3 (6.8)	7 (5.7)
Type of anesthesia				
GA	42 (79.2)	16 (59.2)	0 (0)	58 (46.7)
Spinal	11 (20.8)	11 (40.8)	41 (93.1)	63 (50.8)
Sedation	0 (0)	0 (0)	3 (6.9)	3^a^ (2.5)
Time to set up				
Mean (SD)	5 (1)	9 (2)	9 (2)	7.41 (3.01)
Op time				
Mean (SD)	99 (45)	83 (24)	43 (22)	75.3 (42.05)
Conversion				
Conversion to open surgery	6 (11.5)	4 (14.8)	4 (9.1)	14 (11.4)
Conversion to traditional laparoscopic surgery	2 (3.8)	9 (33.3)	0 (0)	11 (8.9)
No conversion occurred	44 (84.6)	14 (51.9)	40 (90.9)	98 (79.7)
Length of hospital stay				
Mean (SD)	4.4 (2.3)	3.3 (0.8)	1.2 (0.6)	3 (2.1)

*SPF*  semi-private facility, *PF*  private facility, *CHC*  community health center

^a^One patient required sedation and spinal

### Complication rates

One postoperative death was recorded following a laparoscopic cholecystectomy complicated by pancreatitis and sepsis. Overall, 7 (5.7%) patients were reported to have intraoperative complications during cholecystectomy due to bleeding: 2 mild, 4 moderate, and 1 had mild abdominal wall hematoma due to traction. Postoperative complications were reported among 7 (5.7%) patients: 1 diagnostic laparoscopy—1 mild wound infection, 3 gasless cholecystectomies—1 mild injury to structures, 1 moderate wound infection, 1 severe sepsis, 1 Tubal ligation—1 mild wound infection, 2 Other category: 1 appendicectomy and ovarian cystectomy—mild wound infection and 1 gasless conversion to open cholecystectomy and biliary bypass—mild wound infection.

### Conversion

**Fig. 3:**
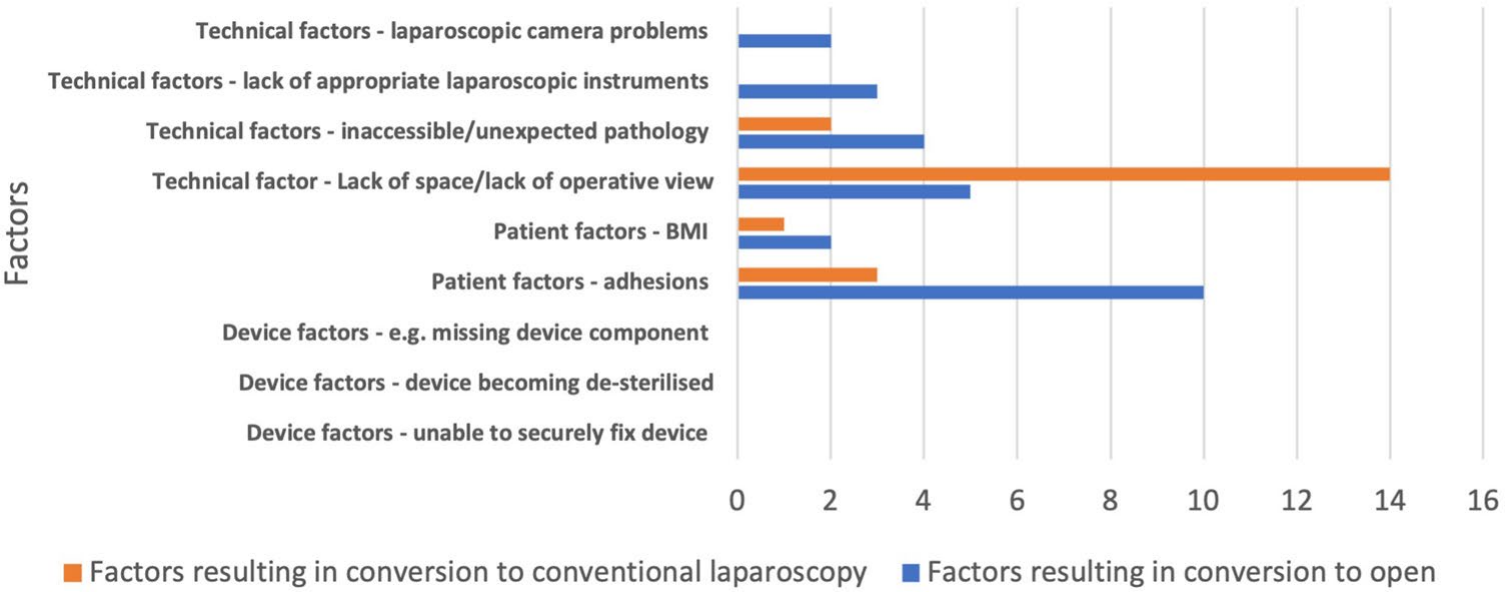
Factors contributing to conversion from gasless to open or conventional laparoscopy

The conversion rate from gasless to open surgery was 11.4%, and 8.9% to conventional laparoscopy. Conversions to open surgery or conventional laparoscopy were primarily due to technical and patient factors. Technical factors leading to conversion to conventional laparoscopy or open surgery included failure to secure a satisfactory operative view and lack of operating space. Adhesions were the leading cause of conversion to open surgery. Device factors were not recorded as a cause for conversion (Fig. 3).

### CUSUM learning curve

Cholecystectomy was the most frequently performed operation, 54/123 (43.9%), followed by tubal ligation, 30/123 (24.3%), and then appendicectomy, 20/123 (16.3%). Most cholecystectomies were performed in Assam, and all tubal ligations were performed in Nagaland. 58/123 (46.7%) patients had a general anesthetic, and 63/123 (50.8%) had a spinal anesthetic. The average operative time for the surgeon who performed most gasless cholecystectomies in Assam was 99 (45) minutes, and in Manipur was 83 (24) minutes.

**Fig. 4:**
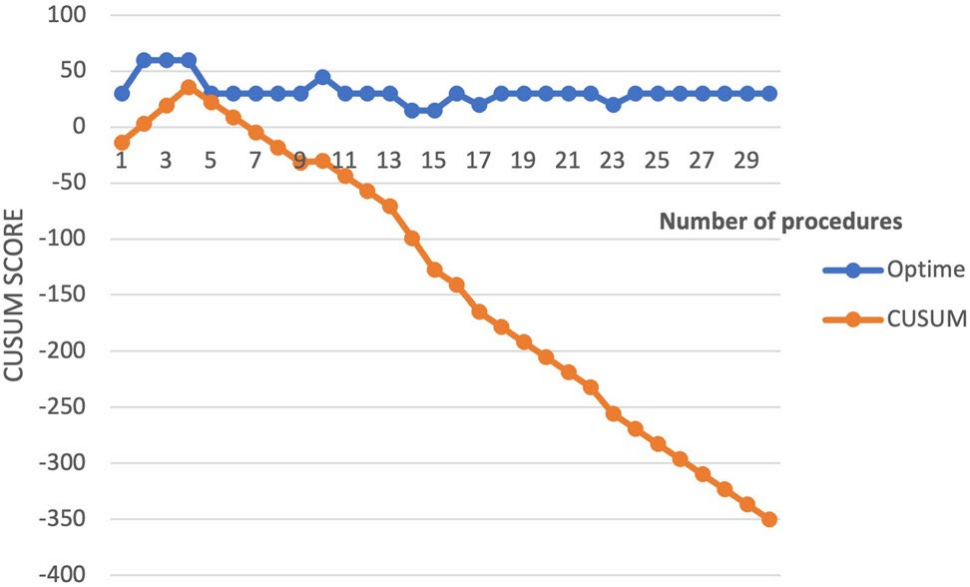
Fixed reference CUSUM learning curve for operative times for gasless tubal ligation by surgeon 2 x-axis = number of procedures. y axis = CUSUM scores/operative time

The CUSUM curve for operative time using the mean score for tubal ligation performed by the surgeon from Nagaland showed steady improvement in operative times after the 4^th^ procedure. Figure 4 demonstrates CUSUM modeling for operating time using a fixed average score of 43.5 min from a published article on gasless tubal ligation [20].

**Fig. 5:**
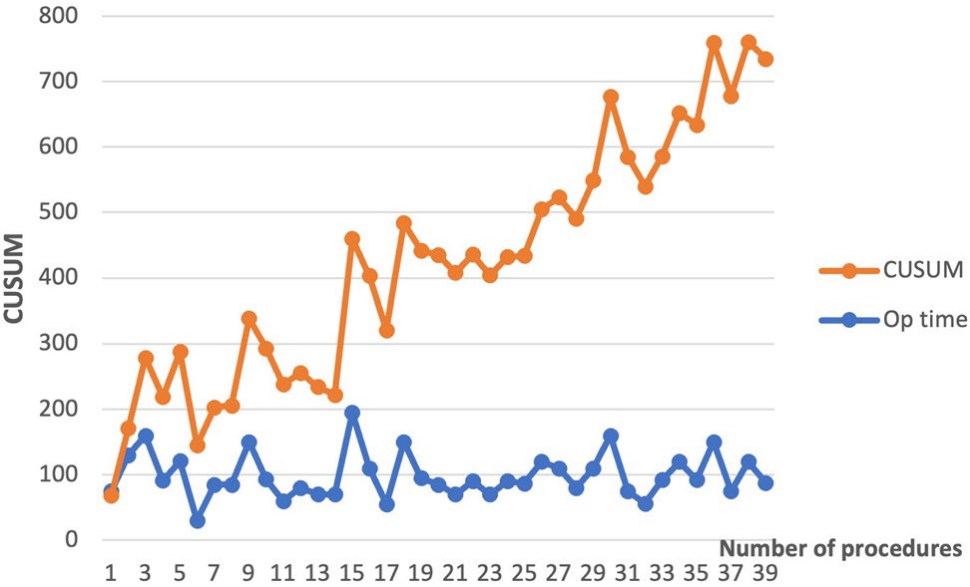
Fixed reference CUSUM learning curve for operative times for gasless Cholecystectomy surgeon 6 who performed most procedures in the registry. x-axis = number of procedures. y axis = CUSUM scores

The CUSUM learning curve for the operative time for gasless cholecystectomy was evaluated for the surgeon from Assam against a fixed average score of 82 min from the literature. This showed a long learning curve for gasless cholecystectomy (Fig. 5).

**Fig. 6:**
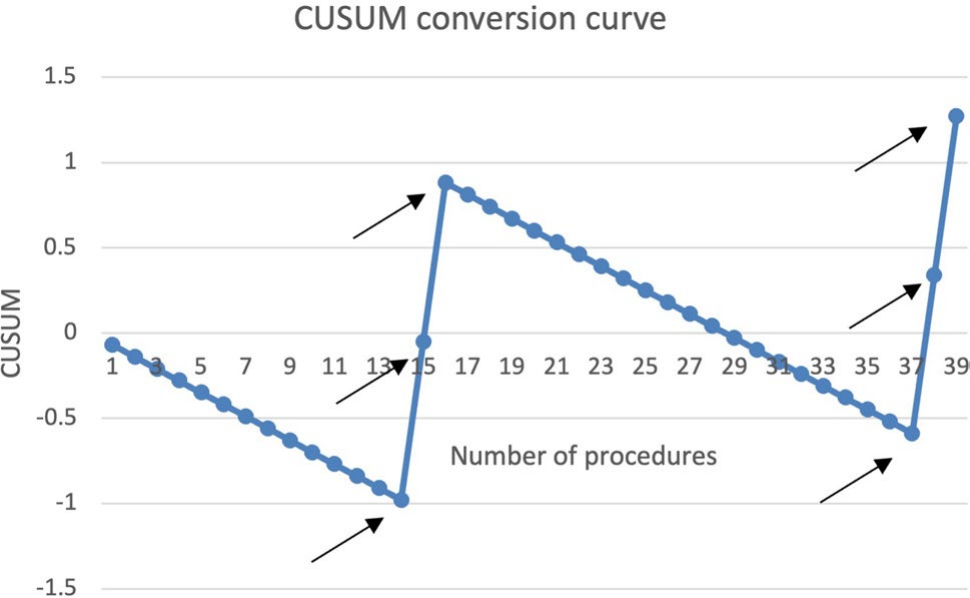
CUSUM learning curve for conversion during gasless Cholecystectomy for surgeon 6, who performed most procedures in the Registry. X-axis = number of procedures. y axis = CUSUM scores. The CUSUM modeling has been performed using a fixed conversion rate of 7% from an RCT. Black arrows indicate the point of conversion

In Fig. 6, the CUSUM learning curve for gasless cholecystectomy was assessed for conversion rates and was compared to a fixed reference conversation rate of 7% [7]. The curve showed that the surgeon in Assam showed some improvement after converting two procedures to open after the 14th case.

### Cost

The mean patient payment for GL cholecystectomy was $201.6 (129.3), and for the hospital stay, $256.2 (77.2). GL appendicectomy was $105.3 (103.6), and the whole hospital stay was $125.9 (29.1). The government fully funded the tubal ligation procedure as part of the family planning initiative (Table 3 Supplement).

## Discussion

The overall intraoperative and post-operative complication rate of 5.7% for GL during the hospital stay compares favorably to the literature. Patients undergoing GL Cholecystectomies experienced intraoperative bleeding at 4.8%: classed as mild to moderate. One patient developed mild abdominal wall hematoma. These patients did not develop post-operative complications during the hospital stay. Overall, 5.7% of patients developed post-operative complications where surgical site infection (SSI) was observed most: 5(4%) classed as mild and one patient was graded moderate after cholecystectomy for cholelithiasis. These incidences are relatively lower compared to previous studies on SSI in limited resource settings after gastrointestinal surgeries [21]. In a phase II non-inferiority single center RCTs in Delhi comparing a total of 100 patients undergoing GL vs conventional laparoscopic cholecystectomy and appendicectomy, the incidence of surgical site infection was 10%, no major complications were recorded, and no statistically significant difference was observed [7]. In a meta-analysis of GL for general surgical and gynecological procedures, we did not observe a difference in intra or postoperative complication rates between gasless and conventional laparoscopy. A subgroup analysis for studies conducted in LMIC showed no difference in outcomes [11]. One patient developed postoperative pancreatitis and sepsis after gasless cholecystectomy for cholelithiasis. A preoperative ultrasound scan showed a single gallstone with normal liver function tests. Post-procedural MRCP did not reveal CBD stones or stricture, demonstrating a cystic duct stump and minimal collection in the gallbladder fossa and perihepatic region. This could be due to passed stones, resulting in pancreatitis and sepsis.

The mean length of stay was three days, comparable to the Delhi RCT of 2.62 days. Patients who underwent cholecystectomy stayed slightly longer, with an average of four days. For appendicectomy, it was three days and one day for tubal ligation. Several hospitals recruited in the study accept state or private insurance policies, making it mandatory for the patient to stay for at least 3 days before discharge. In our review, a shorter stay was observed in patients undergoing GL gynecological procedures when compared to conventional and open surgeries [11].

Costs for procedures such as diagnostic laparoscopy and appendicectomy were relatively lower compared to ovarian cystectomy and cholecystectomy. Each hospital has a standard tariff for procedures, and costs tend to rise if the procedure is performed using conventional laparoscopy due to the costs associated with anesthesia, specialist workforce, and consumables. Complications and extended hospital stay directly affect the payments made by patients for surgical procedures, especially if open or converted to open. Government insurance schemes such as PM-JAY—a national health protection scheme for poor and deprived rural families are available in some centers, which will reimburse a standard amount for a procedure and payments do not change based on a laparoscopic or open technique for some procedures. The package SG017 price for appendicectomy is 11,000 rupees (134 USD), whereas the package SG039 for cholecystectomy is 22,8000 rupees (278 USD)—lap or open [22]. The packages are similar to the costs incurred by patients in this registry. The economic analysis conducted by our group has shown that the scale-up of laparoscopic techniques using the GL technique is a cost-effective alternative to open surgeries for selective essential surgical procedures in North-East India [12]. The latter study is based on the data from 12 healthcare facilities demonstrates that scaling up gasless laparoscopic services would reduce the cost burden to patients and increase DALYs averted if procedures amenable to laparoscopic surgery were performed instead of open technique, which was the standard of care in these facilities.

The lack of working space and achieving satisfactory views are some of the main reasons for conversion to either conventional laparoscopy or open surgery. Time taken to familiarize with the technique, context, and competency in performing GL procedures with limited laparoscopic experience could contribute to the conversion.

The CUSUM learning curve assessing operative time for gasless cholecystectomy showed a long learning curve compared to operatives times used as a fixed reference score from previously published operative time in the Cochrane systematic review of 82 min and 53 min in the Delhi RCT in 2020 [7]. Continued training and practice are likely required to attain expert-level proficiency and increase confidence in complex procedures during gasless laparoscopy in a resource-constrained setting.

Further, the CUSUM learning curve was evaluated using conversion rate as a binary endpoint variable. A systematic review and meta-analysis comparing gasless to conventional laparoscopy show no difference in the conversion rates in general surgical procedures. However, the conversion rate was higher in patients having gynecological procedures [11]. In the Delhi RCT, the reasons for conversion were inadequate space and complex anatomy [7]. Continued research on gasless lift devices has shown better usability in the low resource setting and may improve the operative experience and offer a comparable single quadrant field view to that of conventional laparoscopy [23]. Careful selection of patients is essential for better outcomes in the early stages of the learning curve. A greater focus on case mixes, volume and continued laparoscopic training with the help of simulation and proctorship could reduce the length of the learning curve. Procedure-specific advanced laparoscopic training and remote mentorship could help progress toward expert-level performance.

### Strengths and limitations

Conventional laparoscopy is the standard of care in most private hospitals. Several tertiary government hospitals, level 1 district hospitals, and rural hospitals do not have this facility. Often this is due to a lack of anesthetists or the laparoscopy set-up and maintenance. The study was deemed appropriate for the low resource setting and allowed assessment of the safe implementation of the gasless laparoscopy. Data collection was completed by the rural surgeons, which gave access to reliable, real-time data from rural healthcare setups. There is also a slight difference in the laparoscopic services provided in these centers. The surgeons in the Manipur setup were quick to convert to conventional laparoscopy as this service was readily available but also had a relatively longer learning curve for gasless than that of surgeon 6 from Assam. Affordability plays a huge role in centers that caters to private healthcare. Centers like Assam and Nagaland did not have the luxury of resources or anesthetists and were able to offer the gasless lift technique under spinal or Open surgery.

GL procedures such as appendicectomy, cholecystectomy, and tubal ligation are classed as essential surgical procedures according to DCP-3 (Diseases Control Priorities) and prove to be cost-effective and improve access and quality of surgical care for patients in low-resource settings [24]. The included procedures were based on the expert recommendations of our Indian senior authors and also based on the laparoscopic skills acquisition of the rural surgeons who participated at the TARGET training program and proctorship [9]. As also covered in our systematic review on gasless laparoscopy, given the initial learning curve, limited exposure to laparoscopy and availability of resources, we recommend diagnostic procedures or simple, single-quadrant resection, such as appendicectomy, cholecystectomy, salpingectomy, tubal ligation, oophorectomy, and myomectomy and in non-obese patients with lower anesthetic risk [11]. Tubal ligation is an elective procedure, and using gasless laparoscopy and sterile instruments, we propose a safer approach to sterilization. Practices can be varied in rural healthcare centers, and based on the suggestions of our local experts, gasless tubal ligation was deemed one such procedure that our rural surgeons could perform laparoscopically earlier in the learning curve. More complex procedures can be adopted based on the surgeon’s clinical expertise; however, they fall outside the current objectives of this study.

Inclusion criteria excluded patients who were morbidly obese and/or with multiple co-morbidities; hence findings cannot be generalized to a broader population. The unexpected impact of COVID-19 meant that some surgeons could not perform gasless procedures regularly, which may have affected the proficiency and learning curve. Initially, we were interested in calculating the Risk-Adjusted—CUSUM assessing the difference in conversion rates of gasless laparoscopy between cumulative expected and actual conversion to open or conventional laparoscopy. However, when multivariate logistic regression analysis was conducted (for gasless laparoscopic cholecystectomy or tubal ligation), no independent predictors of conversion were identified to assist with the RA-CUSUM, probably due to the small number of patients included. Procedure-specific learning curve comparisons between surgeons were deemed unsuitable due to a smaller sample size in other centers.

## Conclusion

This study demonstrates the safe implementation of gasless laparoscopy for selective essential abdominal conditions in rural settings in North-East India. Overall outcomes are comparable to the published literature; however, longer operative times, conversion rates, and long learning curves for gasless cholecystectomy require continued training and volume of work to achieve proficiency.

### Supplementary Information

Below is the link to the electronic supplementary material.

Supplementary file1 (DOCX 15 kb)

Supplementary file2 (DOCX 14 kb)
