# Supplementary material for: Gasless laparoscopy in rural India-registry outcomes and evaluation of the learning curve
Source: Surg Endosc. 2023 Aug 31;37(11):8227–35. doi: 10.1007/s00464-023-10392-4 (PMC10615921; doi:10.1007/s00464-023-10392-4)
Supplement: Supplementary file 1 — Supplementary file1 (DOCX 15 kb) [file 464_2023_10392_MOESM1_ESM.docx]

|  | Cases | Mean (SD) Payment for surgery in USD | Mean (SD) Payment for hospital stay | Mean (SD) total payment for entire stay |
| --- | --- | --- | --- | --- |
|  |  |  |  |  |
| Adhesiolysis | 1 (0.8) | 0 | 0 | 0 |
| Appendicectomy | 20 (16.3) | 105.3 (103.6) | 146.5 (186.1) | 125.9 (29.1) |
| Cholecystectomy | 54 (43.9) | 201.6 (129.3) | 310.8 (198.1) | 256.2 (77.2) |
| Diagnostic laparoscopy | 5 (4.1) | 122.3 (107.8) | 171.5 (188.2) | 146.9 (34.8) |
| Other | 6 (4.9) | 155.1 (132.2) | 216.7 (291.3) | 185.9 (43.5) |
| Ovarian cystectomy | 7 (5.7) | 161.1 (87.8) | 275.2 (172.2) | 218.2 (80.6) |
| Tubal ligation | 30 (24.3) | 0 | 0 | 0 |
| OVERALL | 123 | 127.3 (130.9) | 193.5 (212.0) | 160.4 (46.7) |
|  |  |  |  |  |

Table 3 (Supplement): Gasless Registry payment for surgery and hospital stay by procedures. Data are mean and standard deviation (SD)
