# Supplementary material for: Gasless laparoscopy in rural India-registry outcomes and evaluation of the learning curve
Source: Surg Endosc. 2023 Aug 31;37(11):8227–35. doi: 10.1007/s00464-023-10392-4 (PMC10615921; doi:10.1007/s00464-023-10392-4)
Supplement: Supplementary file 2 — Supplementary file2 (DOCX 14 kb) [file 464_2023_10392_MOESM2_ESM.docx]

|  | **Cholecystectomy** | **Tubal Ligation** | **Dx Laparoscopy** | **Others** | **Total** |
| --- | --- | --- | --- | --- | --- |
|  | Intra-operative complications | | | | |
| **Bleeding** |  |  |  |  |  |
| Mild | 2 (1.6) |  |  |  | 2 (1.6) |
| Moderate | 4 (3.2) |  |  |  | 4 (3.2) |
| Severe |  |  |  |  |  |
| **Haematoma** |  |  |  |  |  |
| Mild | 1 (0.8) |  |  |  | 1 (0.8) |
| Moderate |  |  |  |  |  |
| Severe |  |  |  |  |  |
| **Total** |  |  |  |  | 7 (5.7) |
|  | Post-operative complications | | | | |
| **Wound infection** |  |  |  |  |  |
| Mild |  | 1 (0.8) | 1 (0.8) | 2 (1.6) | 4 (3.2) |
| Moderate | 1 (0.8) |  |  |  | 1 (0.8) |
| Severe |  |  |  |  |  |
| **Injury to structures** |  |  |  |  |  |
| Mild | 1 (0.8) |  |  |  | 1 (0.8) |
| Moderate |  |  |  |  |  |
| Severe |  |  |  |  |  |
| **Sepsis** |  |  |  |  |  |
| Mild |  |  |  |  |  |
| Moderate |  |  |  |  |  |
| Severe* | 1 (0.8) |  |  |  | 1 (0.8) |
| Mortality* | 1 (0.8) |  |  |  | 1 (0.8) |
| **Total** |  |  |  |  | 7 (5.7) |

Table 2 (Supplement): Number of Complications - Intraoperative, postoperative and mortality breakdown according to the procedure. Values in parentheses are percentages. Classification EPCO - Mild, Moderate and Severe. Others: 1 procedure lap converted to open cholecystectomy and biliary bypass, 2 gasless ovarian cystectomy + appendicectomy. * Same patient
